# Measures of effective population size in sea otters reveal special considerations for wide‐ranging species

**DOI:** 10.1111/eva.12642

**Published:** 2018-05-17

**Authors:** Roderick B. Gagne, M. Timothy Tinker, Kyle D. Gustafson, Katherine Ralls, Shawn Larson, L. Max Tarjan, Melissa A. Miller, Holly B. Ernest

**Affiliations:** ^1^ Wildlife Genomics and Disease Ecology Laboratory Department of Veterinary Sciences University of Wyoming Laramie Wyoming; ^2^ Western Ecological Research Center U.S. Geological Survey Santa Cruz California; ^3^ Center for Conservation Genomics Smithsonian Conservation Biology Institute Washington District of Columbia; ^4^ The Seattle Aquarium Seattle Washington; ^5^ Department of Ecology and Evolutionary Biology University of California Santa Cruz Santa Cruz California; ^6^ Marine Wildlife Veterinary Care and Research Center California Department of Fish and Game Santa Cruz California

**Keywords:** conservation genetics, effective population size, *Enhydra lutris nereis*, evolutionary potential, genetic monitoring

## Abstract

Conservation genetic techniques and considerations of the evolutionary potential of a species are increasingly being applied to species conservation. For example, effective population size (*N*_e_) estimates are useful for determining the conservation status of species, yet accurate estimates of current *N*_e_ remain difficult to obtain. The effective population size can contribute to setting federal delisting criteria, as was done for the southern sea otter (*Enhydra lutris nereis*). After being hunted to near extinction during the North Pacific fur trade, the southern sea otter has recovered over part of its former range, but remains at relatively low numbers, making it desirable to obtain accurate and consistent estimates of *N*_e_. Although theoretical papers have compared the validity of several methods, comparisons of estimators using empirical data in applied conservation settings are limited. We combined thirteen years of demographic and genetic data from 1,006 sea otters to assess multiple *N*_e_ estimators, as well as temporal trends in genetic diversity and population genetic structure. Genetic diversity was low and did not increase over time. There was no evidence for distinct genetic units, but some evidence for genetic isolation by distance. In particular, estimates of *N*_e_ based on demographic data were much larger than genetic estimates when computed for the entire range of the population, but were similar at smaller spatial scales. The discrepancy between estimates at large spatial scales could be driven by cryptic population structure and/or individual differences in reproductive success. We recommend the development of new delisting criteria for the southern sea otter. We advise the use of multiple estimates of *N*_e_ for other wide‐ranging species, species with overlapping generations, or with sex‐biased dispersal, as well as the development of improved metrics of genetic assessments of populations.

## 1 INTRODUCTION

The benefits of using genetic analyses to inform species conservation and recovery are well known (Allendorf, Luikart, & Aitken, 2012; Frankham, 2010); however, the application of molecular techniques and the implementation of genetic considerations into recovery plans remains limited (Frankham, 2010; Frankham et al., 2017; Pertoldi, Bijlsma, & Loeschcke, 2007; Ralls et al., 2017). Molecular techniques provide the opportunity to assess current genetic status (e.g., genetic diversity, effective population size), detect historic demographic events (e.g., bottlenecks, colonization events; Allendorf et al., 2012; Crandall, Bininda‐Emonds, Mace, & Wayne, 2000), assess population structure (e.g., management units, barriers to gene flow), and identify isolated populations in need of genetic rescue (Frankham et al., 2017). The theory and techniques to address the majority of these considerations are well established. However, unlike more established genetic methods, algorithms for calculating current effective population size (*N*_e_) are still being developed and refined (Do et al., 2014; Waples, Antao, & Luikart, 2014), have stringent model assumptions (Wang, 2016; Waples et al., 2014), and different methods can result in different estimates (Baalsrud et al., 2014; Menéndez, Álvarez, Fernandez, Menéndez‐Arias, & Goyache, 2016; Stubberud et al., 2017). *N*_e_ is usually much less than the census population size (*N*_c_); however, the extremely low *N*_e_/*N*_c_ ratios reported for some species (e.g., marine fishes) are controversial (Waples, 2016). Thus, the implementation of *N*_e_ estimates into applied conservation requires determining the accuracy and feasibility of various *N*_e_ estimators.

Although simple measures of genetic diversity are important for species conservation, *N*_e_ is potentially a more standardized measure that allows target values of genetic diversity to be incorporated into conservation planning (Fisher, 1930; Frankham, Bradshaw, & Brook, 2014; Kamath et al., 2015; Waples, 2002; Wright, 1931). For example, maintaining an *N*_e_ > 500 is thought to be important for long‐term species persistence and adaptability (Frankham et al., 2014). In the simplest sense, *N*_e_ is an estimate of the number of individuals in the population that are contributing genetically, similar to the number of breeding adults (Hedrick, 2011), and is thought to be directly related to population adaptability and viability (Charmantier & Garant, 2005; Frankham et al., 2014; Kamath et al., 2015).

An excellent system for evaluating the consistency of various methods for estimating *N*_e_ is the southern sea otter (*Enhydra lutris nereis*). This population is considered to be a keystone species because of its role in maintaining nearshore marine kelp forests and estuarine sea grasses (Estes & Duggins, 1995; Hughes et al., 2013). It originally ranged from Baja California to Washington State, but was hunted to near extinction during the fur trade. The southern sea otter was thought to be extinct until 1938 when a remnant population of approximately 50 individuals was discovered off Big Sur, California (Leatherwood, Harrington‐Coulombe, & Hubbs, 1978). Over the past decades, the biology of the species and the expansion of the population have been well documented (Ralls, Ballou, & Brownell, 1983; Ralls, Demaster, & Estes, 1996; Tinker et al., 2006; Tinker, Doak, & Estes, 2008b; Tinker et al., 2017). In addition, the southern sea otter is one of the few populations for which the recovery plan considers *N*_e_. The US Fish and Wildlife Service set a delisting criterion of 3,090 otters (as measured by an index consisting of the 3‐year running average of annual range‐wide censuses), a number which was thought to be sufficient to maintain an *N*_e_ of 500 even if large losses were to occur from a major oil spill (Ralls, Demaster, et al., 1996; US Fish and Wildlife Service, 2003). We obtained this criterion using a multiplier of 3.7 to convert *N*_e_ to census size (*N*), so that an *N*_e_ of 500 corresponds to 1,850 individuals (Ralls, Demaster, et al., 1996). However, in more than 20 years since the plan was written, long‐term field studies and advancements in molecular and computational techniques have facilitated more precise *N*_e_ estimates (Ralls et al., 1983; Ralls, Demaster, et al., 1996; Wang, 2016; Waples, Do, & Chopelet, 2011), and the recovery plan recommends reevaluating the *N*_e_ calculations when better estimates are possible (US Fish and Wildlife Service, 2003).

Questions about population status are especially timely for southern sea otters because the population index has exceeded the 3,090 threshold for the last 2 censuses (Tinker & Hatfield, 2017; Tinker & Hatfield, 2016), and delisting consideration will be triggered if the index exceeds the threshold for a third year (US Fish and Wildlife Service, 2003). The population has increased in both abundance and distribution over the past several decades, with the current range extending along the mainland from Gaviota State Park in the south to Pigeon Point in the north, as well as San Nicolas Island in the southern California Bight (Tinker & Hatfield, 2017). Nevertheless, there are still contemporary threats to the population including the potential for an oil spill, increased mortality from lethal shark bites (Tinker, Hatfield, Harris, & Ames, 2016), as well as deaths from pathogens and toxicants associated with freshwater runoff (Jessup et al., 2007; Miller et al., 2010; Tinker et al., 2016). In light of these continued threats, the impending delisting decision, and the availability of improved *N*_e_ estimators, a re‐investigation of *N*_e_ estimates is needed. Consideration of genetic factors is especially important for species, such as the sea otter, that have undergone extreme bottleneck events which can cause long‐term reductions in genetic diversity and result in inbreeding depression (Aguilar, Jessup, Estes, & Garza, 2008; Larson, Jameson, Etnier, Jones, & Hall, 2012).

Although multiple *N*_e_ estimators have been developed since the recovery plan was written, use of each method for a specific application requires careful consideration of its underlying assumptions and consistency with other methods (Baalsrud et al., 2014; Wang, 2016). For example, accurate estimates of *N*_e_ rely upon a thorough understanding of population structure (Hössjer, Laikre, & Ryman, 2016). Determining genetic structure can be complicated by a number of factors that influence spatial patterns of allele frequencies, such as sex‐biased dispersal (Garant, Dodson, & Bernatchez, 2001; Handley & Perrin, 2007). The effects of sex‐biased dispersal on *N*_e_ is a concern for sea otters because males can roam throughout the range of the subspecies, but sexually mature females exhibit strong site fidelity (Lafferty & Tinker, 2014; Ralls, Eagle, & Siniff, 1996; Tarjan & Tinker, 2016). Furthermore, *N*_e_ can be calculated using genetic or demographic data. To date, demographic and genetic *N*_e_ estimates have rarely been calculated on the same population, and when they have, there were inconsistencies between demographic and genetic *N*_e_ estimates (Baalsrud et al., 2014; Stubberud et al., 2017; Waples et al., 2011).

We genotyped 1,006 southern sea otters sampled over 13 years at 38 microsatellite markers and combined those results with long‐term demographic data to address the question of the consistency of *N*_e_ estimators and their usefulness for updating the southern sea otter recovery plan. We first determined the genetic structure of southern sea otters and evaluated temporal changes in genetic diversity in order to properly parameterize estimators of *N*_e_ and to provide additional assessments of sea otter genetic variation. We then used the information gained from our genetic analyses and our demographic data to parameterize and run multiple *N*_e_ estimators to appraise their consistency. These analyses will inform management and recovery strategies for southern sea otters. In addition, the comparison between genetic and demographic *N*_e_ estimates will guide a diverse group of stakeholders including conservation biologists, wildlife managers, and policymakers, to the potential difficulties of each method, the utility of conducting multiple independent analyses, and the issues with obtaining estimates across large geographic areas.

## 2 METHODS

### 2.1 Demographic data

We collected the data required for demographic estimation of *N*_e_ during population studies conducted throughout the sea otter’s range in California between 2000 and 2014 (Tinker, Bentall, & Estes, 2008a; Tinker et al., 2006, 2017). These studies involved the capture, tagging, and subsequent monitoring (via radio telemetry over 3–5 year periods) of over 350 individual sea otters. We computed the estimates of age at first reproduction and age‐specific survival and reproductive rates from these data using maximum likelihood and Bayesian‐based mark–recapture models, as described elsewhere (Tinker et al., 2006, 2017). We estimated the variation in female reproductive success (defined as the annual probability of producing and successfully weaning a pup) from the individual histories of over 100 females (Staedler, 2011) and the variation in male reproductive success (defined as the relative contribution to paternity of surviving pups in each cohort) from a genetic paternity analysis of 67 males and 183 pups (Tarjan, 2016).

### 2.2 Sample collection

We obtained skeletal muscle samples from sea otter carcasses recovered through a large‐scale stranding network conducted by the California Department of Fish and Wildlife (CDFW), the US Geological Survey (USGS), the Monterey Bay Aquarium (MBA), and The Marine Mammal Center (TMMC) (Kreuder et al., 2003). We obtained additional archival blood, hair, and buccal swab samples from live‐sampled sea otters captured as part of ongoing mark–recapture studies conducted by USGS in conjunction with CDFW and MBA (Tinker et al., 2006, 2017). Sea otters were aged in the field using standard tooth eruption, tooth wear, external morphometrics and pelage characteristics, as previously described (Tinker et al., 2006). For stranded carcasses and a subset of live animals, age estimates were cross‐validated using cementum analysis of sampled premolar teeth (Bodkin, Ames, Jameson, Johnson, & Matson, 1997). We calculated the birth date for individual otters by subtracting the estimated age at capture/carcass recovery from the capture or necropsy date. Genomic DNA was isolated from 10 to 20 mg tissue or 100 to 200 μl blood using QIAGEN DNeasy Blood & Tissue kit (QIAGEN Inc., Valencia, CA, USA). For hair and swab samples, we extracted DNA using the QIAamp DNA Micro Kit.

### 2.3 Genotyping

A panel of 38 microsatellite loci was used to genotype sea otters at the University of California Davis, Ernest Lab and Veterinary Genetic Laboratory. We obtained microsatellite loci and methods for amplification from Larson, Jameson, Etnier, Fleming, and Bentzen (2002), Kretschmer, Olsen, and Wenburg (2009), and Arias et al. (2016) and are further described in supplemental materials. All genotypes were run and confirmed twice, and each plate of DNA included both positive and negative controls. Two people separately used STRand Analysis Software (Toonen & Hughes, 2001) to score and bin alleles to ensure consistent allele calls. MS toolkit (Park, 2001) was used to access potential duplicate samples, and suspected duplicates were removed from the dataset.

We conducted the evaluations of loci to test for deviations from Hardy–Weinberg proportions (HWP) and linkage disequilibrium in *Genepop* v4.2 (Rousset, 2008). We performed sequential Bonferroni corrections on *p*‐values to account for multiple comparisons (Holm, 1979). In addition, we conducted tests for deviation from HWP on cohorts of sea otters and present the uncorrected results. We calculated the number of alleles, *F*_IS_, Shannon information index, and expected and unbiased expected heterozygosity using *GenAlEx* v6.501 (Peakall & Smouse, 2012). We calculated allelic richness, which controls for differences in sample sizes, in *R* 3.2.1 (R Core Development Team, 2013) using the package “PopGenReport” (Adamack & Gruber, 2014). We compared unbiased heterozygosity and allelic richness across year of capture and year of birth to evaluate changes in genetic diversity over time using a linear model implemented in *R*.

### 2.4 Genetic structure

We assessed genetic structure in three ways: grouping all samples by county of collection, using specific location for a subset of individuals with available GPS data for their capture or carcass retrieval location, and observation‐based home range for the smaller subset of tagged otters (Tarjan & Tinker, 2016). Analyses of otters with known home ranges were included as this information most accurately reflects the location in which an otter lived, instead of where it happened to be captured or washed ashore. We evaluated genetic structure for sea otter samples originating from north of Santa Cruz County, Santa Cruz + Monterey Counties, San Luis Obispo County, south of San Luis Obispo County, and San Nicolas Island using Bayesian clustering software *Structure v2*.3.4 (Pritchard, Stephens, & Donnelly, 2000, supplemental materials). We also determined genetic structure using discriminant analysis of principal components (DAPC) (Jombart, Devillard, & Balloux, 2010) run in *R* (R Core Development Team, 2013), with package “adegenet” (Jombart, 2008). Because dispersal in sea otters is sex‐biased (Lafferty & Tinker, 2014; Ralls, Eagle, et al., 1996; Tarjan & Tinker, 2016), we conducted DAPC and *Structure* analyses separately for each sex, as well as with a combined dataset.

Spatially explicit Bayesian analyses that incorporated GPS location data were run using the program *TESS* 2.3 (Durand, Chen, & Francois, 2009). *TESS* software models population admixture proportions over a multidirectional surface while incorporating spatially autocorrelated random effects in the form of a conditional autoregressive residual term. This serves to model broad‐scale genetic clustering across a multidirectional surface while adjusting for genetic structure associated with relatedness between individuals and isolation by distance (Durand, Chen, & Francois, 2009). The package “POPSutilities” in *R* was used to map sampling locations with the population assignment probabilities (*Q* coefficients) throughout the southern sea otter range (Jay et al., 2012). We conducted all genetic structuring analyses on both pooled and sex‐specific datasets. Test for genetic isolation by distance was run using Rousset’s estimate (Rousset, 2000) implemented in “Genepop” (Rousset, 2008) for all samples with GPS coordinates (*n* = 712). In addition, we evaluated geographic genetic variation using the single population spatial autocorrelation implemented with 999 permutations in *GenAlEx* v6.501 (Peakall & Smouse, 2012). The analyses considered twenty‐nine distance classes of 25 km determined using geographic distances between individuals.

### 2.5 Effective population size

We calculated the genetic estimates of effective population size (*N*_e_) using the linkage disequilibrium information (Hill, 1981), implemented in *NeEstimator* V2.01 (Do et al., 2014) and the sibship frequency method, implemented in the program *Colony* V2.0 (Jones & Wang, 2010). These methods are the most robust and accurate single sample genetic estimators of *N*_e_, and the two‐sample methods are not applicable because our data do not span a sufficient number of generations (i.e., at least 3–5 for species with overlapping generations) (Waples & Yokota, 2007). To access changes in *N*_e_ over time, we conducted the linkage disequilibrium (LD) method on two subsets of otters with known or estimated birth dates between 1995 and 2000, and between 2000 and 2005. In addition, by implementing the LD method on cohorts of otters, we estimated the number of breeders (*N*_b_) across years. We also calculated the estimates of *N*_e_ and *N*_b_ from demographic life table data using the program *AgeNe* (Waples et al., 2011). *AgeNe* software allows for two sexes with unequal sex ratio and/or differential survival, and can also account for deviances from Poisson variance in reproductive success (Waples et al., 2011). We provide detailed explanations of input variables and calculations used with *AgeNe* (Waples et al., 2011) in the supplementary material.

We conducted the estimates of *N*_e_ across the entire southern sea otter range, as preliminary genetic estimates suggested there is no population structure within the range. However, because tagging studies show that females otters disperse relatively short distances (Tarjan & Tinker, 2016; Tinker et al., 2006, 2008b), there is the potential of isolation by distance. This would affect *N*_e_ estimates, so we repeated all calculations for a geographic subset of the population, limiting analysis to otters from Monterey County.

## 3 RESULTS

### 3.1 Genetic diversity

**Figure 1:**
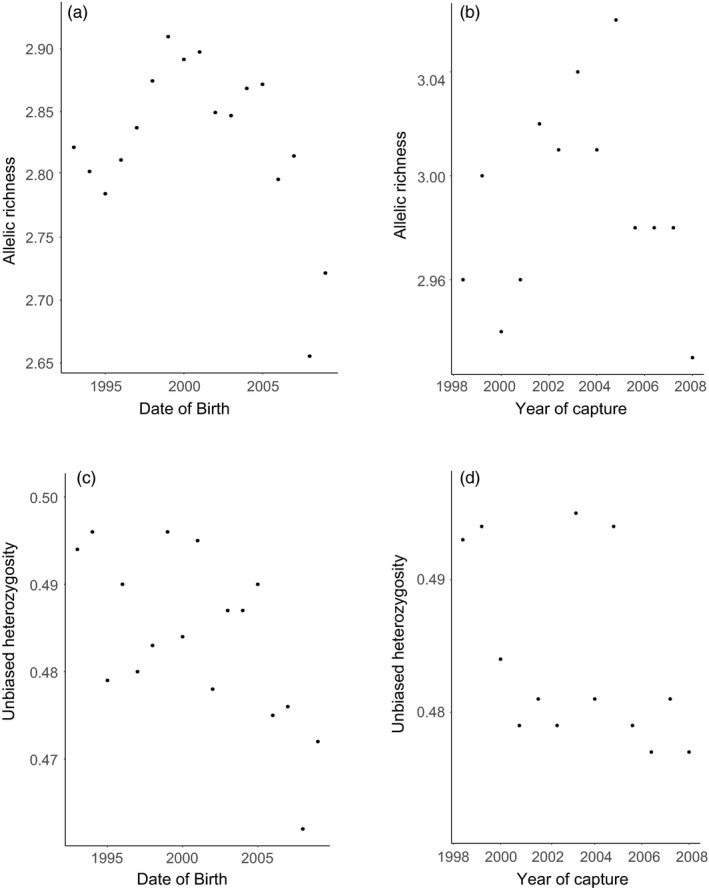
Mean measures of genetic diversity for southern sea otters (*Enhydra lutris nereis*), including allelic richness (a) across the estimated year of birth and (b) the year of sample collection as well as unbiased expected heterozygosity (c) across the estimated year of birth and (d) the year of sample collection. Both metrics have a slight but significant decrease with the year of birth

We obtained genotypes for approximately one‐third of the contemporary population size (~3,000) of the southern sea otter, totaling 1,006 individuals, at 38 microsatellite loci, including 819 for which a birthdate could be estimated, 712 individuals where the GPS location of capture/carcass recovery was known, and 176 radio‐tagged animals with observation‐based home range estimates. Table 1 presents the genetic diversity metrics for all samples and across collection year. Inbreeding levels (*F*_IS_) were 0.16 across the entire range and 0.011 for the otters in Monterey County. Evaluating a subset of samples from a single year and location revealed no loci that violated Hardy–Weinberg proportions (HWP). However, when analyzing across all samples, ten of the 38 loci violated HWP, and tests for deviations of HWP by cohort found 60 of 887 tests violated HWP (Supporting information Table S1). Significant linkage disequilibrium was found in only seven of 703 comparisons (<1%). There were no significant deviations in genetic diversity across year of capture but the unbiased expected heterozygosity slightly but significantly decreased with year of birth (*T* = −3.08, *df* = 16, *p* = 0.007, Figure 1, Supporting information Table S1).

**Table 1:** Mean measures of genetic diversity in southern sea otters (*Enhydra lutris nereis*) over a 13‐year period, including the sample size (*N*), number of alleles (*N*_a_), allelic richness (*A*_r_), Shannon’s information index (*I*), observed heterozygosity (*H*_o_), expected heterozygosity (*H*_e_), and unbiased expected heterozygosity (u*H*_e_)

Year collected	*N*	*N*_a_	*A*_r_	*I*	*H*_o_	*H*_e_	u*H*_e_
All	1,006	4.5	3.0	0.86	0.49	0.50	0.50
1998	16	3.1	3.0	0.82	0.48	0.48	0.49
1999	38	3.3	3.0	0.84	0.49	0.49	0.49
2000	27	3.1	2.9	0.82	0.47	0.48	0.48
2001	72	3.3	3.0	0.83	0.48	0.48	0.48
2002	86	3.4	3.0	0.83	0.48	0.48	0.48
2003	156	3.5	3.0	0.83	0.47	0.48	0.48
2004	84	3.4	3.0	0.86	0.48	0.49	0.50
2005	67	3.4	3.0	0.83	0.47	0.48	0.48
2006	79	3.5	3.1	0.86	0.48	0.49	0.49
2007	79	3.4	3.0	0.83	0.48	0.48	0.48
2008	73	3.3	3.0	0.83	0.47	0.47	0.48
2009	74	3.3	3.0	0.83	0.46	0.48	0.48
2010	35	3.2	2.9	0.81	0.49	0.47	0.48

Only years with at least ten samples are presented in individual Year collected rows, while the All row includes all samples in the dataset.

### 3.2 Population genetic structure

**Figure 2:**
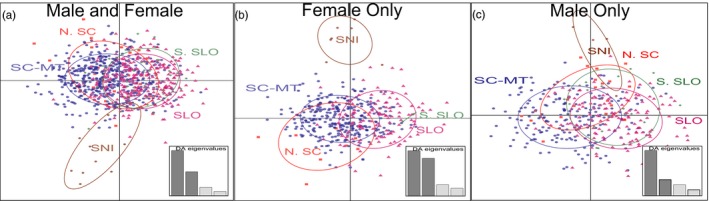
Results of discriminant analysis of principal components (DAPC) of southern sea otters grouped by major California coastal region: North of Santa Cruz County (N.SC), Santa Cruz and Monterey counties (SC/MT), San Luis Obispo County (SLO), south of San Luis Obispo County (S.SLO), and San Nicolas Island (SNI) for (a) mixed sex, (b) females only, and (c) males only. Insert graph represents the eigenvalues of the first four discriminant functions, with the dark gray bars identifying the discriminant functions being presented

Genetic *Structure* analyses revealed no significant genetic structure across geographic regions for the entire dataset and when analyzing each sex separately (Supporting information Figure S1). These results were unchanged when 11 individuals from San Nicolas Island (SNI) were included. *Structure Harvester* indicated *K* = 1, and structure output graphs at *K* = 2 and *K* = 3 both revealed a clear lack of genetic structure (Supporting information Figure S1). The DAPC analysis revealed that female SNI otters clustered together, but no other clustering was observed (Figure 2). Tests for isolation by distance (IBD) were significant, but IBD explained only a small amount of genetic variation (*r* ^2^ = 0.002, *p* = 0.008). MSA analysis shows minimal influence of distance on genetic differences except some minimal effects at the largest spatial scales (Supporting information Figure S2).

**Figure 3:**
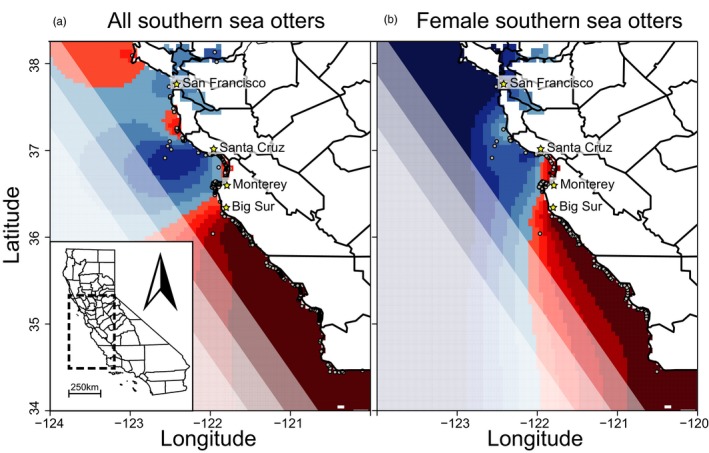
Map of posterior estimates of admixture proportions for sampled southern sea otters (*Enhydra lutris nereis*) calculated using *TESS*. The color corresponds to the assigned genetic cluster with the shade of color corresponding to the *Q* value or proportion assignment to that cluster (lightest shade represents *Q* values 0.5–0.6, darkest shade *Q *>* *0.9). Each dot represents a sampled otter, and color of genetic cluster extends into the ocean but otters are near shore species. Analyses were run for the combined dataset with pooled males and females (a) and females only (b)

Spatially explicit *TESS* models for females did not find a clear number of clusters following the Bayesian information criterion. The graphical output, however, reveals a clear genetic break corresponding to otters sampled within and north of Monterey County, compared with those from south of Monterey (Figure 3). The assignment proportions in *TESS* indicated greater assignment to a genetic cluster as distance increased from the spatial convergence of these two groups (Figure 3). Similarly, *TESS* models for pooled males and females did not find a clear number of clusters, but assigned otters in Monterey County to a cluster (Figure 3). No spatial genetic structure was detected through analysis of animals with known home ranges (which typically span just 5–25 km of coastline), even though these individuals were distributed across the entire range of the population (Tarjan & Tinker, 2016).

### 3.3 Effective population size

Estimates of *N*_e_ varied across methods of calculation (Table 2). For genetic estimates, the linkage disequilibrium method found an *N*_e_ of 320 (95% CI 297–344) and the sibship frequency method found an *N*_e_ of 485 (95% CI = 424–550). Although *N*_e_ was greater in otters born between 2000 and 2004 than for otters born between 1995 and 1999, the confidence intervals overlapped extensively (Table 2) and there was no trend in the number of breeders across years (Supporting information Table S1). Demographic estimates for effective population size (*N*_e_ = 1,230) across the entire population were more than double the genetic estimates (*N*_e_ LD = 320, *N*_e_ SF = 485); however, demographic and genetic estimates were consistent for otters in Monterey County (Table 2). In comparison, the census population size at the end of our sampling period (2012) was 2,792 total sea otters, or 2,469 excluding dependent pups (Tinker & Hatfield, 2016).

**Table 2:** Estimates of the effective population size for southern sea otters (*Enhydra lutris nereis*) based on multiple estimators

Sample	Method	*N*_e_	95% CI
All	Linkage Disequilibrium	341	287–410
	Sibship frequency	485	424–550
	Demographic *N*_e_	1,230	1,087–1,272
	Demographic *N*_b_	1,103	596–1,401
Monterey	Linkage Disequilibrium	200	180–223
	Sibship frequency	243	203–293
	Demographic *N*_e_	278	245–287
	Demographic *N*_b_	247	133–314
Born 1995–1999	Linkage Disequilibrium *N*_b_	302	225–430
Born 2000–2004	Linkage Disequilibrium *N*_b_	334	251–470

The effective population size (*N*_e_) and 95% confidence intervals (95% CI) are listed. Analyses utilized a 13‐year dataset encompassing 1,006 sampled individuals (All), and subsets of otters from Monterey, with known or estimated birth dates between 1995 and 1999, and 2000 and 2004. Demographic analyses include the effective population size (*N*_e_) and the number of breeding individuals (*N*_b_), based on 2012 total abundance estimates.

## 4 DISCUSSION

Our findings provide insight into the consistency of genetic estimators of *N*_e_, the importance of direct monitoring of genetic diversity, and unanswered questions concerning southern sea otters. We found little evidence for population genetic structure, and sea otter genetic diversity remained consistent throughout the study period, despite a large increase in sea otter population size. The latter finding suggests that current management practices will likely not result in substantially increased genetic diversity, which must occur via mutations in a closed population. The discrepancies between genetic and demographic *N*_e_ range‐wide estimates, but similarity within a single region, suggest cryptic genetic structure may be affecting *N*_e_ estimates at large geographic scales despite minimal evidence for apparent genetic structure. This finding has implications for other threatened or endangered long‐lived species with a wide geographic range, in which accurate estimates of *N*_e_ would be useful for proper management but may be difficult to obtain.

### 4.1 Genetic structure

We found no evidence of population differentiation across the entire range of southern sea otters, which is consistent with previous work (Aguilar et al., 2008), suggesting that conservation efforts targeted at preserving the population as a whole are well founded. However, managing a species across a large geographic area poses some challenges. In order to reduce the chance of an oil spill decimating the entire population, sea otters were translocated to San Nicolas Island (SNI) between 1987 and 1990 from the mainland California coastal population; however, most of the original animals dispersed back to the mainland (a minimum distance of 110 km) or are unaccounted for Hatfield (2005). Our female‐only analyses revealed that otters from the surviving population at SNI are now genetically distinct from all other California sea otters. In contrast, no genetic structure was found for SNI males, although the sample size was small. As long‐range dispersal from the island to the mainland is possible, our findings suggest that males may occasionally disperse from the mainland to San Nicolas. It is difficult, however, to differentiate contemporary dispersal from genetic similarities as a result of mainland otters serving as the source for introductions to SNI.

Although we found no discrete subpopulations along the California mainland, we did find weak but statistically significant isolation by distance suggesting geographically distant sea otters have greater genetic differences but gene flow occurs across the range of the subspecies. When evaluating only female sea otters, the *TESS* analysis revealed a split in genetic groups near Big Sur, with genetic differences increasing further away from this Monterey/Big Sur split. These observations suggest genetic isolation by distance extending north and south from Big Sur, instead of linearly from north to south down the entire range. This pattern is consistent with the geographic pattern of recovery and range expansion over the last century, described as diffusion northwards and southwards from the original remnant population in Big Sur (Lubina & Levin, 1988; Tinker et al., 2008b). Furthermore, population bottlenecks followed by subsequent range expansion can result in allelic surfing, where rare alleles become more common on the range edges resulting in different allele frequencies in the newly occupied territories (Excoffier, Matthieu, & Petit, 2009; Hofer, Ray, Wegmann, & Excoffier, 2009). Allelic surfing could explain some of the fine‐scale genetic structure and the lack of linear isolation by distance.

### 4.2 Effective population size

Demographic and genetic estimates of *N*_e_ were consistent when comparing a subset of the population from Monterey County (approximately 1/3 of the total population), but the range‐wide genetic estimates of *N*_e_ were much lower than demographic estimates. Estimates of *N*_e_ can vary between computational techniques, by sample size, and with the number of loci used, which makes accurately estimating *N*_e_ for wildlife populations difficult (Wang, 2016; Waples, 2016). Moreover, estimates of *N*_e_ will be inaccurate if taken across a genetically structured area (Wang, 2016). We were able to overcome many of these obstacles by sampling a large number of individuals, incorporating multiple estimators (Kamath et al., 2015), and by analyzing more than 20 microsatellite loci (Wang, 2016). Despite these precautions, we still found an inconsistency in range‐wide estimates of *N*_e_. The robust genetic and demographic datasets make it unlikely that lack of data or insufficient genetic markers are influencing the results.

The consistency of demographic and genetic estimates within a portion of the population, but not across the range, suggests that the discrepancies between demographic and genetic estimates of *N*_e_ over the entire range could be caused by cryptic genetic structure and isolation by distance (Ryman, Allendorf, Jorde, Laikre, & Hössjer, 2014). When the range of a population spans a distance greater than that over which dispersal and mating normally occur, genetic estimates of *N*_e_ can be lowered due to the pooling of geographically separated individuals with different allele frequencies. This occurs because estimators of *N*_e_ assume that differences in allele frequencies are caused predominantly by genetic drift (Neel et al., 2013). The presence of cryptic genetic structure is consistent with findings from multiple tagging studies, which indicate minimal dispersal of animals between the northern portion of the range and the southern portion of the range (Tarjan & Tinker, 2016; Tinker et al., 2008b, 2017). Females in particular rarely move more than 25 km from their home range center; however, some adult males are more mobile, occasionally moving throughout the entire range. A few migrants per generation can remove signals of genetic structuring at neutral markers, even if groups are predominantly isolated, resulting in the inability to detect genetic structure but enough variation in allele frequencies across the range to bias genetic *N*_e_ estimates (Allendorf, Hohenlohe, & Luikart, 2010; Neel et al., 2013; Ryman et al., 2014). Furthermore, we measured demographic data across the range and showed limited geographic variation in reproductive success and survival (Tinker et al., 2006, 2017), making it unlikely that erroneous parameterization would affect the range‐wide estimates but not the Monterey County estimates. These differences between demographic and genetic estimates of *N*_e_ reveal limitations of current techniques in accurately estimating *N*_e_ across large areas.

Age structuring of populations can bias estimates of *N*_e_ in a variety of ways that differ between estimators. Genetic estimators that are based on the assumption of discrete generations and sampling across multiple generations can result in underestimates of the *N*_e_ by 10%–50% (Waples et al., 2014). Typically, this is a result of linkage disequilibrium caused by the admixture of parents of different age mating and creating a two‐locus Wahlund effect (Nei & Li, 1973; Waples et al., 2014). In contrast, the consistent individual differences in lifetime reproductive success of sea otters, which would decrease the true *N*_e_ (Lee, Engen, & Sæther, 2011; Stubberud et al., 2017), are not accounted for in demographic estimates of *N*_e_ resulting in a potential overestimate (Lee et al., 2011; Staedler, 2011; Stubberud et al., 2017; Tarjan, 2016). We suggest that the combined effects of isolation by distance over a large geographic area, the influence of overlapping generations, and consistent individual variation in reproductive success, explain the difference between genetic and demographic range‐wide estimates of *N*_e_. However, the consistency of genetic and demographic estimates within a single geographic area (Monterey County) suggests that cryptic structure across the sea otter range may be the primary cause for the discrepancies in methods.

Few prior studies have examined genetic and demographic estimates of *N*_e_ within a single system, and those that have found contrasting results to ours. For example, in house sparrows on islands, it was found that genetic estimates of *N*_e_ were higher than demographic estimates (Baalsrud et al., 2014). In that study, however, there were multiple populations with evidence of gene flow among populations, increasing genetic *N*_e_ estimates (Baalsrud et al., 2014). Thus, the house sparrow example represents the inverse scenario to our current study, in which there is a single population with cryptic structure that could not be detected by our analyses. The differences in *N*_e_ between demographic and genetic estimates, and the inconsistencies of these differences, highlight the importance of using multiple lines of evidence when possible.

### 4.3 Genetic diversity

We found low levels of genetic diversity when compared to other sea otter populations (*H*_e_ range 0.48 to 0.86; Larson et al., 2012; this study *H*_e_ 0.50), with no significant change over the 13‐year study period, and a decrease in genetic diversity when grouping otters by birth year. Although confirmation of consistent genetic diversity over the sampling period is promising, increased diversity would be beneficial to recover what was lost during the bottleneck associated with fur trade hunting (Larson et al., 2002, 2012). Gene flow via translocations, re‐introductions, and natural dispersal can increase genetic diversity (Weeks et al., 2011). California populations, however, have had no documented introductions or migrations from the northern sea otter subpopulation, and the geographic distance between them currently exceeds the natural dispersal distance for sea otters (Larson, Bodkin, & VanBlaricom, 2015). Another factor that could increase genetic diversity would be an increase in the rate of population growth (Ortego, Calabuig, Cordero, & Aparicio, 2007). However, given the multiple threats impacting the southern sea otter population (including disease and shark bites), and because range expansion has stalled over the past two decades due to increased mortality at the northern and southern range peripheries (Lafferty & Tinker, 2014; Tinker & Hatfield, 2016; Tinker et al., 2016), it seems unlikely that demographic factors will enhance genetic diversity in the immediate future. Our findings also reveal that an increase in population size did not result in a corresponding increase in genetic diversity, suggesting that population size is not always a suitable substitution for direct measurements of genetic diversity, particularly for closed populations that have experienced severe population bottlenecks (Frankham, 2010). The study period, however, only encompassed the time span of one or two generations (generation time of 7.9 years, estimated from life history table) and, due to lack of gene flow with northern sea otters, any increases in diversity would have to be driven by mutations, thus our sampling period may be too short to detect increases in genetic diversity.

### 4.4 Conservation implications

Long‐term analyses suggest that genetic diversity in southern sea otters is not increasing despite the continued, though sluggish, increase in population size. As sea otters from Washington State are gradually expanding into Oregon, eventual reestablishment of connectivity with California populations would seem to be a real possibility. However, the cessation of range expansion to the north by southern sea otters has so far precluded recolonization of the coastline north of San Francisco Bay. Should the northern and southern sea otter populations eventually merge, it would increase the genetic diversity of the CA population, potentially improving health and increasing adaptive potential.

Our findings highlight fundamental difficulties in defining clear indicators of the genetic status of a population or species (Pierson, Luikart, & Schwartz, 2015). In our case, the discrepancies in genetic and demographic estimates of *N*_e_ are problematic in terms of providing a simple *N*_e_‐based metric to support decisions about management and conservation of southern sea otters. Our results suggest that the genetic estimates of *N*_e_ at the range‐wide scale are too low because of the problem of applying genetic estimates over a large geographic range in which there may be cryptic genetic structure due to isolation by distance. If we instead use the demographic estimate of *N*_e_, and account for the potential bias introduced by differences in lifetime reproductive success (a 10%–50% reduction), we estimate that the current *N*_e_ of the southern sea otter population is between 544 and 1,145. Because *N*_e_ is not increasing with the growth of the population, and because we are unable to provide a single precise estimate of the number of otters corresponding to an *N*_e_ of 500 as required by the recovery plan, we conclude that the current delisting criterion is not appropriate for southern sea otters. Instead, approaches such as population viability analyses that can incorporate genetic and demographic factors to determine extinction risks could serve to better inform delisting decisions: Such an integrated approach can incorporate the importance of maintaining sufficient genetic variation while encompassing additional factors (Benson et al., 2016). In addition, *N*_e_ itself can be incorporated into recovery plans using it as a risk element as a part of a more comprehensive plan (Allendorf et al., 1997). The planning process for Pacific salmon recovery included consideration of *N*_e_ to some extent in nine of eleven recovery plans, for example in setting minimum abundance levels. The plans also include multiple delisting criteria which ensure that the important aspects of *N*_e_ are considered but delisting is not based on a specific value of *N*_e_ without considering other important factors (C. Busack, NOAA Fisheries, Portland Regional Office, personnel communication; e.g., Hard et al., 2015; Williams et al., 2008).

We found good agreement between the measures of effective population size within a subset of the range, indicating that *N*_e_ can be effectively estimated when the assumptions of the analyses are met. Inconsistencies between the methods across the entire range of the sea otter population, however, revealed the difficulties of accurately evaluating *N*_e_ for long‐lived species with large geographic distributions despite no apparent genetic structure. Demographic analyses of *N*_e_ may not be possible for some species because acquiring demographic data is expensive, time‐consuming, and requires many years of continued monitoring. In contrast, genetic estimates are effective for small, isolated populations (Wang, 2005), as is the case for many species of conservation concern. However, our results suggest these estimates often may be problematic for species that, even at low numbers, tend to have larger ranges and longer life spans. Advances in sequencing technology permit the inclusion of thousands of loci in estimates of *N*_e_ but this does not increase precision of estimates (Waples, Larson, & Waples, 2016), indicating that continued research is needed on methods to obtain better estimates of contemporary *N*_e_ for wide‐ranging mammal species of conservation concern. For sea otters, as for many threatened populations, multiple independent estimates may be the best approach.

## CONFLICT OF INTEREST

None Declared.

## DATA ARCHIVING STATEMENT

Microsatellite genotypes have been deposited and made publically available on Dryad https://doi.org/10.5061/dryad.j992pv8.

Demographic data used in effective population size estimates are included in the supplemental materials and references cited therein.
