# Supplementary material for: Measures of effective population size in sea otters reveal special considerations for wide‐ranging species
Source: Evol Appl. 2018 May 17;11(10):1779–90. doi: 10.1111/eva.12642 (PMC6231473; doi:10.1111/eva.12642)
Supplement: Supplementary file 1 [file EVA-11-1779-s001.pdf]

Supplemental material

Table S1. The table shows (N) the number of sampled otters estimated to be born in each year, (Nb) the estimated number of breeders calculated with *NeEstimator,* (HW-#T) the number of loci (i.e., all that were not monomorphic) tested for being in Hardy Weinberg Proportions, and (HW-S) those loci found to significantly deviate from Hardy Weinberg Proportions. The table represents a subset of sea otters with available age estimates.

Date of birth	N	Nb	HW-#T	HW-S
1983	1	-	15	0
1984	6	-	35	0
1985	0	-		
1986	2	-	29	0
1987	1	-	21	0
1988	0	-		
1989	3	-	33	0
1990	1	-	15	0
1991	10	121 (31-Infinite)	38	0
1992	5	-	36	1
1993	23	914 (93-Infinite)	38	3
1994	19	Infinite	38	4
1995	34	366 (144-Infinite)	38	7
1996	49	783 (161-Infinite)	38	2
1997	71	217 (112-1087)	38	3
1998	94	170 (107-353)	38	6
1999	77	1620 (342-Infinite)	38	6
2000	93	435 (235-1992)	38	5
2001	76	290 (165-941)	38	3
2002	84	238 (157-458)	38	2
2003	47	212 (108-1532)	38	2
2004	53	635 (175-Infinite)	38	7
2005	24	404 (96-Infinite)	38	4
2006	11	Infinite	37	2
2007	20	608 (103- Infinite)	38	2
2008	9	-	36	0
2009	4	-	32	1
2010	2	-	28	0

Figure S1. Results from Structure with population groups corresponding to major geographic regions, north of Santa Cruz County (SC), Santa Cruz and Monterrey counties (SC/MT), San Luis Obispo County (SLO), and south of San Luis Obispo County (South of SLO). Individuals are along the x-axis with the amount of each color corresponding the proportion of assignment to that group.

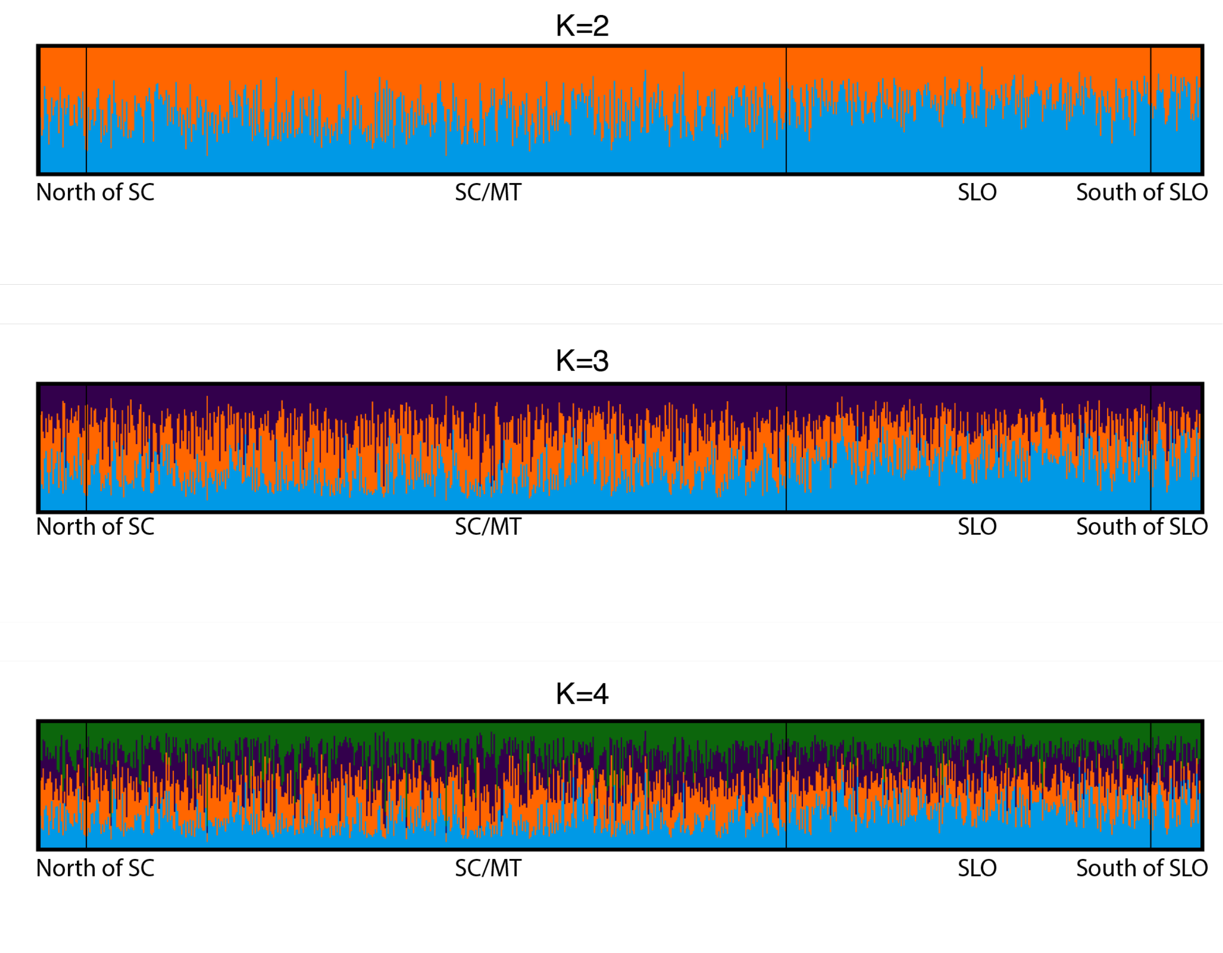

Figure S2. A. Pairwise comparison of individuals geographic distance to linear genetic distance. B. Results of single population spatial autocorrelation. Graph shows correlations in r across distance class sizes of 25km. Error bars represent 95% confidence intervals calculated using bootstrapping resampling. Permutations (999) were used to calculate 95% confidence intervals surrounding the null hypothesis of no spatial structure (represented as red lines on graph).

A.

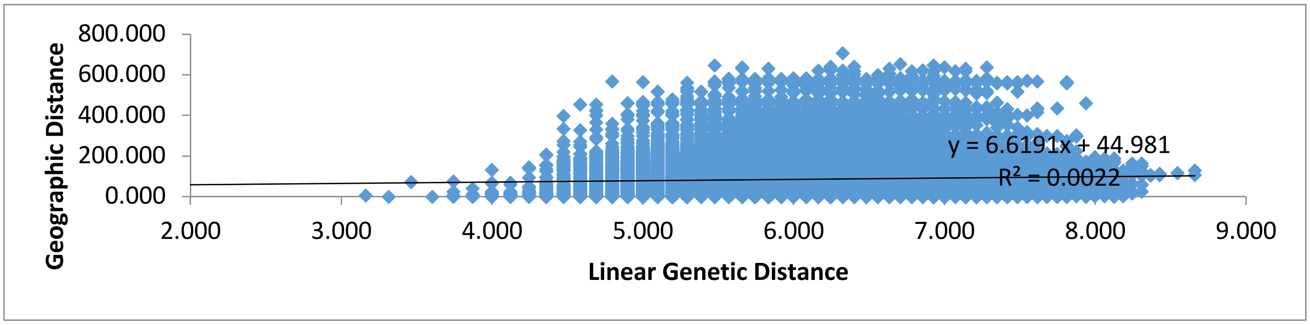

B.

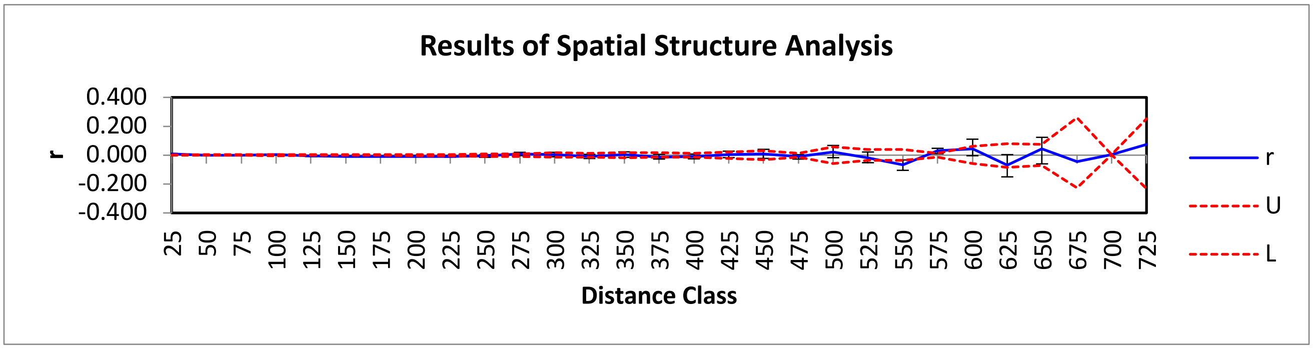

Details for “AgeNe” program

We used the program “AgeNe” (Waples *et al.* 2011) to estimate the effective population size (N_e) from demographic parameters. Input parameters for AgeNe are sex-specific, and include age-specific reproductive output (b_i, defined for females as ½ x birth rate x probability of pup surviving to become an independent juvenile, and for males as ½ x the mean annual number of pups sired that survive to become an independent juvenile), age-specific annual survival rate (s_i), the number of animals entering into the first year-class of independents (N_1), and the “Poisson Variance Inflation Factor” (φ). We define b_i as the probability of producing and successfully weaning a pup (rather than just birth rate) because weaned juveniles represent the first independent age class tracked by the model. This formulation is appropriate for sea otters, as all mature females typically produce a pup every year but there is variance in the ability to wean the pup. Age-specific variance in b_i is therefore primarily a reflection of variation in weaning success. Estimates of b_i, and s_i were available from multiple mark-recapture studies conducted throughout California (Tinker *et al.* 2006; Tinker *et al.* 2017), and we used the range-wide average values to parameterize the input file for AgeNe (Table S2). To compute N_1 we took the product of the estimated population size (independent animals 1 year of age and older, excluding dependent pups) from the 2012 range-wide census (Tinker and Hatfield 2016) and the first element of the stable stage distribution vector (summed for males and females), as computed analytically from a Leslie matrix model population parameterized with b_i, and s_i values. Estimating φ was less straightforward: φ represents the ratio of the variance to the mean of individual reproductive contributions to each cohort, and may vary among ages and between males and females. For female sea otters after the age of first reproduction, virtually all individuals give birth to a single pup per year (Monson *et al.* 2000; Staedler 2011; Tinker *et al.* 2006), while the likelihood of successfully weaning the pup varies as a function of age, so per-capita reproductive output represents a Bernoulli variable with possible outcomes 1 or 0 and probability $p = b_{f,i}$ (Figure S1). Variance in annual reproductive output for females of age i ($V_{f,i}$) is thus described as the variance of a Bernoulli variable, $p(1\text{-}p)$, and we calculated age-specific values of $\varphi_{f,i}$ as:

$$\varphi_{f,i} = {}^{V_{f,i}}\!\big/_{b_{f,i}} \tag{1.1}$$

Note that $V_{f,i}$ only accounts for within-age class variance in reproductive output: consistent year-to-year differences in reproductive success among females (non-random lifetime mating) can also affect N_e (Lee *et al.* 2011) but are not explicitly accounted for by AgeNe.

For males, reproductive output corresponds to the mean expected number of pups sired in a given year. In the special case of random mating, this value could be described as a Poisson variable with mean and variance of $b_{m,i}$. However, recent genetic paternity analyses (Tarjan 2016) indicate that male reproductive success is not random, but varies both by age (Figure S2) and by male status, with almost all surviving pups sired by the fraction of the male population that successfully defends reproductive territories. To account for age-based variation, we computed $b_{m,i}$ from the age-specific frequency of detected paternity’s (dp_i) as described by Tarjan 2015 (Tarjan 2016):

$$b_{m,i} = C\left({}^{dp_i}\!\Big/_{\sum_i dp_i} \right) \tag{1.2}$$

where C is a constant fitted such that $\Sigma(b_{m,i} \text{ x } N_i) = ½N_1$. However, we note that $b_{m,i}$ is averaged

across both successful territorial males (whose average reproductive contribution we define as k_i) and non-territorial males (whose mean reproductive contribution ~ 0). If we specify *f* as the fraction of males contributing to reproduction, then k_i can be calculated as $b_{m,i} \text{ x } f^{1}$, and variance in reproductive success is estimated as:

$$V_{m,i} = f\left(k_i + k_i^2\right) - b_{m,i}^2 \tag{1.3}$$

As with females, we estimate age-specific values of the variance inflation factor ($\varphi_{m,i}$) as:

$$\varphi_{m,i} = {V_{m,i}}/{b_{m,i}} \tag{1.4}$$

Based on the genetic data from the paternity analysis (Tarjan 2016) augmented by radio telemetry data from multiple population studies, we determined that a biologically plausible range of values for *f* was 0.2 to 0.8, with a median value of 0.5. Uncertainty in the value of *f* translated into variation in $\varphi_{m,i}$ (Figure S2); accordingly, to characterize the range of uncertainty attributable to non-random mating by males, we ran AgeNe using high (0.8), low (0.2) and median (0.5) values of *f*. We also ran versions of the model for the entire sea otter range (total number of independent otters N=2,469, number in first year class N_1 = 460) and for Monterey County only (total number of independent otters N=555, number in first year class N_1 = 104). A sample input file (range-wide estimate, median estimate of *f*) is shown in Table S2.

Table S2. Input file for "AgeNE" program (table contents to be saved as a text file). First line provides text summary of file and is ignored by program. Second line consists of 3 parameters: number of year classes, number of individual animals in first year-class, and sex ratio (proportion female). Remaining lines consist of 7 data fields (columns) repeated for each year class (rows): Age of year class, female survival rate, female mean reproductive output, female "Poisson Variance Inflation Factor" (φ_f), male survival rate, male mean reproductive output, male "Poisson Variance Inflation Factor" (φ_m).

Txt: Sea otters range-wide (lambda = 1), male f = 0.5 (MEDIAN SKEW)

18	460	0.5				
1	0.682	0	0	0.662	0	0
2	0.746	0	0	0.778	0	0
3	0.917	0.144	0.856	0.844	0.05	1.05
4	0.965	0.212	0.788	0.883	0.174	1.174
5	0.971	0.291	0.709	0.911	0.405	1.405
6	0.956	0.301	0.699	0.901	0.654	1.654
7	0.896	0.289	0.711	0.871	0.654	1.654
8	0.866	0.271	0.729	0.833	0.521	1.521
9	0.846	0.262	0.738	0.858	0.332	1.332
10	0.817	0.269	0.731	0.873	0.231	1.231
11	0.818	0.287	0.713	0.867	0.12	1.12
12	0.788	0.287	0.713	0.853	0.082	1.082
13	0.755	0.268	0.732	0.8	0.084	1.084
14	0.654	0.237	0.763	0.734	0.068	1.068
15	0.431	0.157	0.843	0.431	0.041	1.041
16	0.201	0.073	0.927	0.201	0.022	1.022
17	0.149	0.054	0.946	0.149	0	0
18	0.142	0.052	0.948	0.142	0	0

Figure S3. Estimates of reproductive output (*b*) as a function of age for female and male sea otters in California, based on monitoring data from radio tagged sea otters. Plotted values for females represent mean expected number of successfully weaned pups per year per female. Plotted values for males represent mean expected number of sired pups surviving to independence, based on genetic paternity analysis. See text for methods.

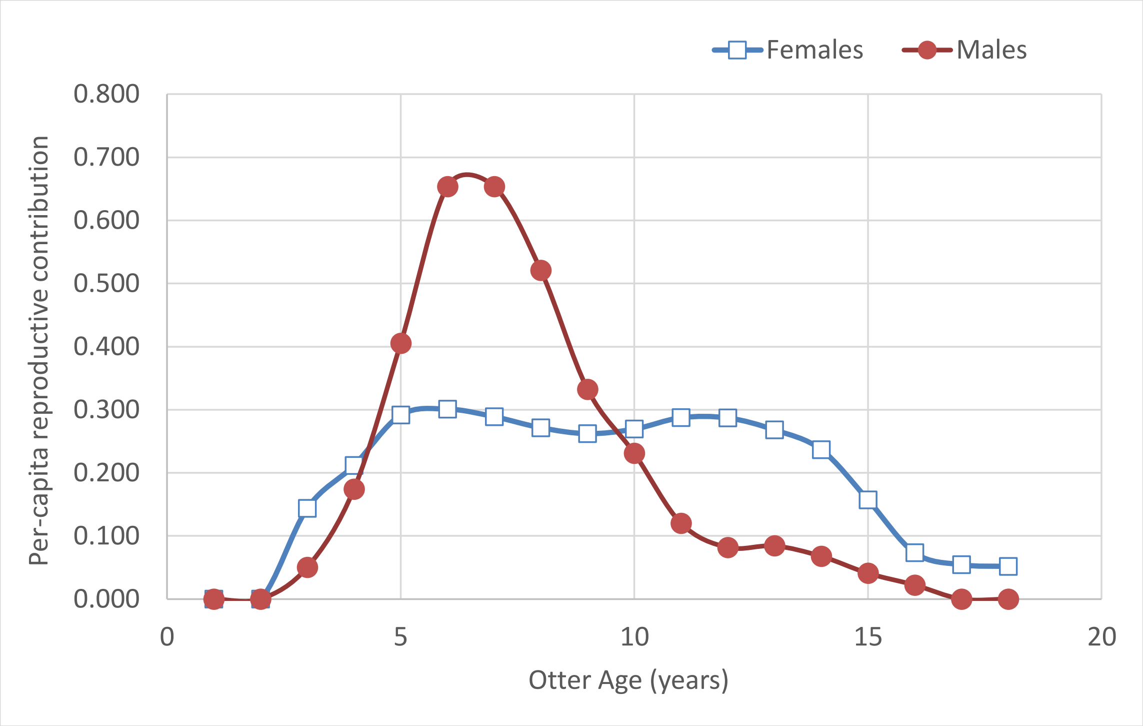

Figure S4. Values of the variance inflation factor for male sea otters plotted as a function of age, for three different levels of *f* (the fraction of males contributing to reproduction).

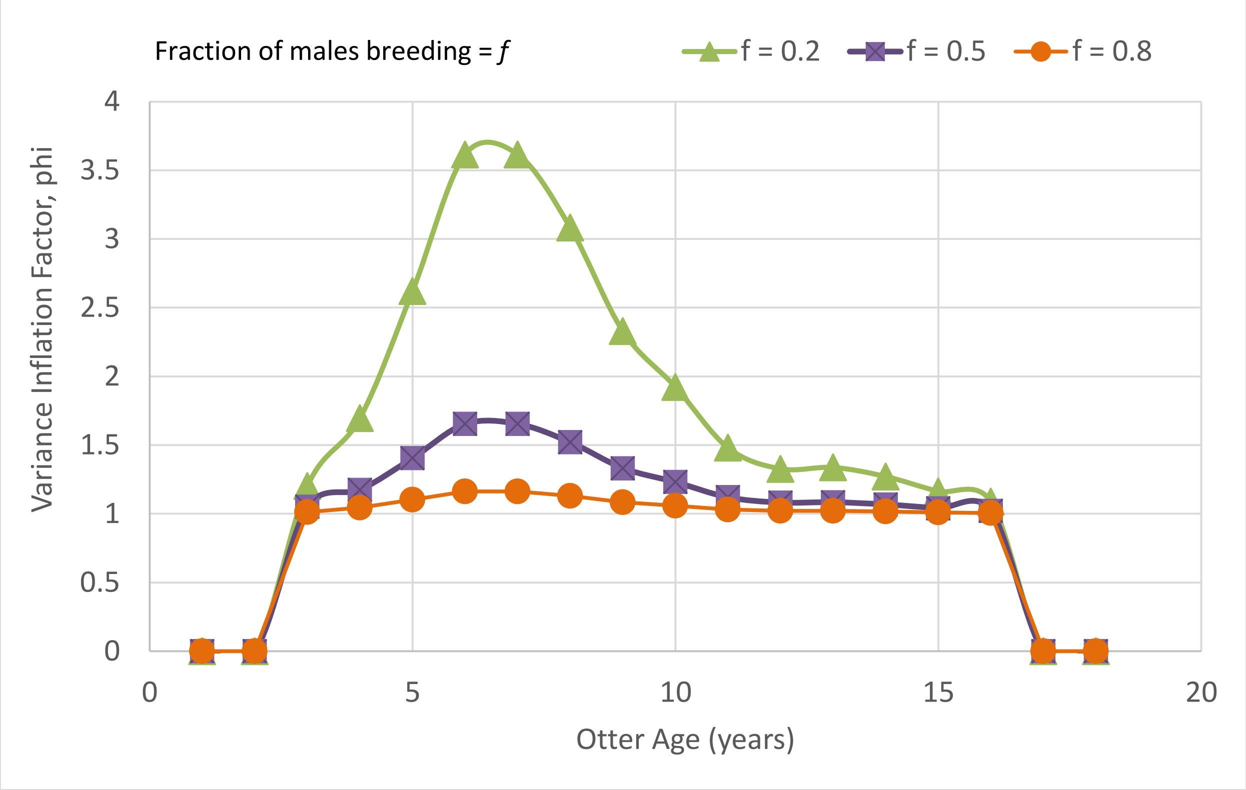

Microsatellite genotyping

Microsatellite loci and methods for amplification were obtained from Larson et al. (2002), Kretschmer et al. (2009), and Arias et al. (2016). Loci were multiplexed prior to polymerase chain reaction (PCR) using QIAGEN Multiplex PCR Kit (QIAGEN). PCRs were done in 12.5µl reactions containing: 6.25µl of 2x QIAGEN multiplex PCR master mix (final concentration, 1x), 1.25ul of primer mix, 1.25ul of 5x Q-solution, 1.75µl of molecular-grade water and 1–2µl of DNA. Amplifications were carried out in a Bio-Rad MyCyler (Bio-Rad, Hercules, CA, USA) with the following conditions: 15 min at 95°C, 40 cycles consisting of 94°C for 30 s, 57°C for 90 s, and 72°C for 90 s, followed by a final extension step of 72°C for 10 min. Forward primers were labeled with a fluorescent dye of NED, PET, FAM or VIC from Applied Biosystems (Life Technologies, Carlsbad, CA, USA) and run on a 3730 DNA Analyzer (Applied Biosystems Inc.) with 0.05 µl GeneScan 500 Liz Size Standard and 9.95 µl of HiDi formamide (Applied Biosystems Inc.).

Structure parameters

The program *Structure* was implemented using the admixture model with correlated allele frequencies (Hubisz *et al.* 2009; Pritchard *et al.* 2000) and allowing the parameter α to vary among populations. We ran 10 replicate analyses of the full dataset with a burn-in period of 100,000 iterations, followed by 500,000 Markov chain Monte Carlo repetitions. Consecutive runs were conducted with K from 1–6 in analysis of geographic regions. *Structure Harvester* (Earl & vonHoldt 2012) was used to determine the maximum level of genetic structure using the rate of change of the log probability of data [lnP(D)] between successive K values (ΔK) following Evanno *et al.* (2005). The final structure plots and Q coefficients for each population were obtained by averaging the 10 runs for K 1–6 in *CLUMPAK* using the default parameters and 2000 random input order repeats (Kopelman 2015).

Literature cited:

Arias MC, Aulagnier S, Baerwald EF*, et al.* (2016) Microsatellite records for volume 8, issue 1. *Conservation Genetics Resources* **1**, 43-81.

Earl DA, vonHoldt BM (2012) STRUCTURE HARVESTER: a website and program for visualizing STRUCTURE output and implementing the Evanno method. *Conservation Genetics Resources* **4**, 359-361.

Evanno G, Regnaut S, Goudet J (2005) Detecting the number of clusters of individuals using the software STRUCTURE: a simulation study. *Molecular Ecology* **14**, 2611-2620.

Hubisz MJ, Falush D, Stephens M, Pritchard JK (2009) Inferring weak population structure with the assistance of sample group information. *Molecular ecology resources* **9**, 1322-1332.

Kopelman NM, Mayzel, J., Jakobsson, M., Rosenberg, N. A., & Mayrose, I. (2015) Clumpak: a program for identifying clustering modes and packaging population structure inferences across *Molecular ecology resources* **15**, 1179-1191.

Kretschmer EJ, Olsen JB, Wenburg JK (2009) Characterization of eight microsatellite loci in Sea Otter, Enhydra lutris, and cross-species amplification in other Mustelidae. *Conservation Genetics* **10**, 775-777.

Larson S, Jameson R, Etnier M, Fleming M, Bentzen P (2002) Loss of genetic diversity in sea otters (Enhydra lutris) associated with the fur trade of the 18th and 19th centuries. *Molecular Ecology* **11**, 1899-1903.

Lee AM, Engen S, Sæther B-E (2011) The influence of persistent individual differences and age at maturity on effective population size. *Proceedings of the Royal Society of London B: Biological Sciences* **278**, 3303-3312.

Monson DH, Estes JA, Bodkin JL, Siniff DB (2000) Life history plasticity and population regulation in sea otters. *Oikos* **90**, 457-468.

Pritchard JK, Stephens M, Donnelly P (2000) Inference of population structure using multilocus genotype data. *Genetics* **155**, 945-959.

Staedler MM (2011) *Maternal care and provisioning in the southern sea otter (Enhydra lutris nereis): reproductive consequences of diet specialization in an apex predator*.

Tarjan LM (2016) *Space Use and Reproductive Success of Male Sea Otters*, U. California Santa Cruz.

Tinker MT, Doak DF, Estes JA*, et al.* (2006) Incorporating diverse data and realistic complexity into demographic estimation procedures for sea otters. *Ecological Applications* **16**, 2293-2312.

Tinker MT, Tomoleoni J, LaRoche N*, et al.* (2017) Southern sea otter range expansion and habitat use in the Santa Barbara Channel, California. US Geological Survey.

Waples RS, Do C, Chopelet J (2011) Calculating Ne and Ne/N in age-structured populations: a hybrid Felsenstein-Hill approach. *Ecology* **92**, 1513-1522.
